# Supplementary material for: Inequity in the face of success: understanding geographic and wealth-based equity in success of facility-based delivery for under-5 mortality reduction in six countries
Source: BMC Pediatr. 2024 Feb 28;23(Suppl 1):651. doi: 10.1186/s12887-023-04387-2 (PMC10900542; doi:10.1186/s12887-023-04387-2)
Supplement: Supplementary file 1 — Additional file 1. [file 12887_2023_4387_MOESM1_ESM.docx]

**Inequity in the face of success: Understanding geographic and wealth-based equity in success of facility-based delivery for under-5 mortality reduction in six countries**

**Additional Files**

**Additional File 1. List of neonatal evidence-based interventions**

| **Period of risk** | **Evidence-based intervention** | |
| --- | --- | --- |
| Preconception | Folic acid supplementation | |
| Antenatal | Tetanus vaccination | |
|  | Malaria prevention and treatment | Intermittent presumptive treatment |
|  |  | Insecticide-treated bed nets |
|  | Iodine supplementation (in endemic iodine deficient settings) | |
|  | 4 or more antenatal visits (ANC4) | |
|  | Prevention and treatment of preeclampsia and eclampsia | Antihypertensive treatment for severe hypertension |
|  |  | Magnesium sulfate |
|  |  | Early delivery |
| Intrapartum | Antibiotics for preterm premature rupture of membranes | |
|  | Corticosteroids for preterm labor | |
|  | C-section for breech or obstructed labor | |
|  | Active management of delivery (including partograph) | |
|  | Clean delivery practices (incl. clean cord-cutting) | |
|  | Trained birth attendant | |
|  | Facility-based delivery | |
|  | Basic emergency obstetric and newborn care (BEmONC) | |
|  | Comprehensive emergency obstetric and newborn care (CEmONC) | |
|  | Timely transport for higher level care for mother | |
| Postnatal | Newborn resuscitation | |
|  | Immediate breastfeeding | |
|  | Prevention and management of hypothermia | Immediate drying and wrapping |
|  |  | Delayed bathing |
|  |  | Skin-to-skin |
|  |  | Baby warming |
|  | Kangaroo care for LBW/prematurity | |
|  | Timely transport for higher level care for mother | |
|  | Post-partum visits to identify danger signs and provide active referral | |
|  | Antibiotics for suspected or confirmed infection | |
|  | Surfactant therapy for respiratory distress syndrome and prematurity | |
|  | Neonatal intensive care units (equipped, trained staff, standards and protocols established and followed) | |
