# Supplementary material for: Inequity in the face of success: understanding geographic and wealth-based equity in success of facility-based delivery for under-5 mortality reduction in six countries
Source: BMC Pediatr. 2024 Feb 28;23(Suppl 1):651. doi: 10.1186/s12887-023-04387-2 (PMC10900542; doi:10.1186/s12887-023-04387-2)
Supplement: Supplementary file 2 — Additional file 2. [file 12887_2023_4387_MOESM2_ESM.docx]

**Additional File 2. Change in under-5 mortality and neonatal mortality in six countries (10–21)**

| **Country** | **Ethiopia** | | **Rwanda** | | **Senegal** | | **Peru** | | **Bangladesh** | | **Nepal** | |
| --- | --- | --- | --- | --- | --- | --- | --- | --- | --- | --- | --- | --- |
|  | 2000 | 2016 | 2000 | 2015 | 1999 | 2015 | 2000 | 2014 | 2000 | 2014 | 2001 | 2016 |
| Under-5 mortality (deaths per 1000 live births) | 166 | 67 | 196 | 50 | 145 | 59 | 60 | 23 | 110 | 30 | 91 | 39 |
| Relative change (%) | **-60** | | **-74** | | **-59** | | **-62** | | **-73** | | **-57** | |
| Neonatal mortality (deaths per 1000 live births) | 58 | 29 | 44 | 19 | 39 | 23 | 23 | 12 | 50 | 14 | 39 | 21 |
| Relative change (%) | **-50** | | **-57** | | **-41** | | **-48** | | **-72** | | **-46** | |
